# Cytomorphology of nodular histiocytic/mesothelial hyperplasia

**DOI:** 10.1002/dc.24979

**Published:** 2022-05-17

**Authors:** Xiaobing Jin, Xin Jing, Jonathan B. McHugh, Liron Pantanowitz

**Affiliations:** ^1^ Department of Pathology University of Michigan Ann Arbor Michigan USA

**Keywords:** cytomorphology, nodular histiocytic/mesothelial hyperplasia

## Abstract

Nodular histiocytic/mesothelial hyperplasia (NHMH) is a pathologic entity that has not been well characterized in the cytopathology literature. This is unfortunate because if unrecognized, NHMH may be misdiagnosed when encountered in cytology specimens. The aim of this communication is to accordingly alert cytologists about NHMH by means of an illustrative case report.

A 51‐year‐old male patient, who never smoked and had no significant past medical history, presented with left sided chest pain. A computerized tomography (CT) scan of his chest revealed a large 13.4 × 11.5 × 16.0 cm heterogeneous pleural‐based mass with an associated small left pleural effusion. There was no metabolic imaging evidence of distant metastatic malignant disease on a positron emission tomography (PET)/CT scan. Excision of the mediastinal mass revealed a completely excised paraganglioma. The pleural fluid (30 ml) was sent for cytological examination. A direct smear preparation, ThinPrep slide and cell block material were prepared from his sample and displayed moderate cellularity. There were large cohesive aggregates of mononuclear cells observed within a background of dyshesive vacuolated histiocytic cells and reactive epithelioid mesothelial cells (Figure 1A,B). A separate population of neoplastic cells was difficult to discern. Cellular aggregates were present on smears and the ThinPrep slide (Figure 1C). The cell block showed compact cellular nodules that were surrounded by a thin layer of fibrin (Figure 1D). These dense nodules contained a heterogenous mix of round and polygonal cells with abundant pale‐pink cytoplasm and only mild nuclear pleomorphism. No mitoses or necrosis was present. Immunohistochemistry performed on cell block material demonstrated that these cellular nodules were predominantly comprised of histiocytes (CD68 and CD163 positive) (Figure 1E) that were enmeshed with scattered mesothelial cells (pancytokeratin, calretinin, WT‐1, Desmin and CK5/6 positive) (Figure 1F). CD34 was positive on the cell membrane of a subset of scattered mesothelial cells (Figure 1G). Histopathological examination of the resected pleural‐based mass revealed a typical paraganglioma with nests of epithelioid cells separated by fibrovascular stroma (Figure 1H). The diagnosis of a paraganglioma was confirmed by immunohistochemistry staining. Negative immunostaining of cells in the cell block material for GATA3, synaptophysin and chromogranin excluded pleural fluid involvement by paraganglioma. All pleural fluid cells were also negative for EMA, Claudin‐4, MOC‐31 and TTF‐1. These findings were compatible with the diagnosis of NHMH.

**FIGURE 1:**
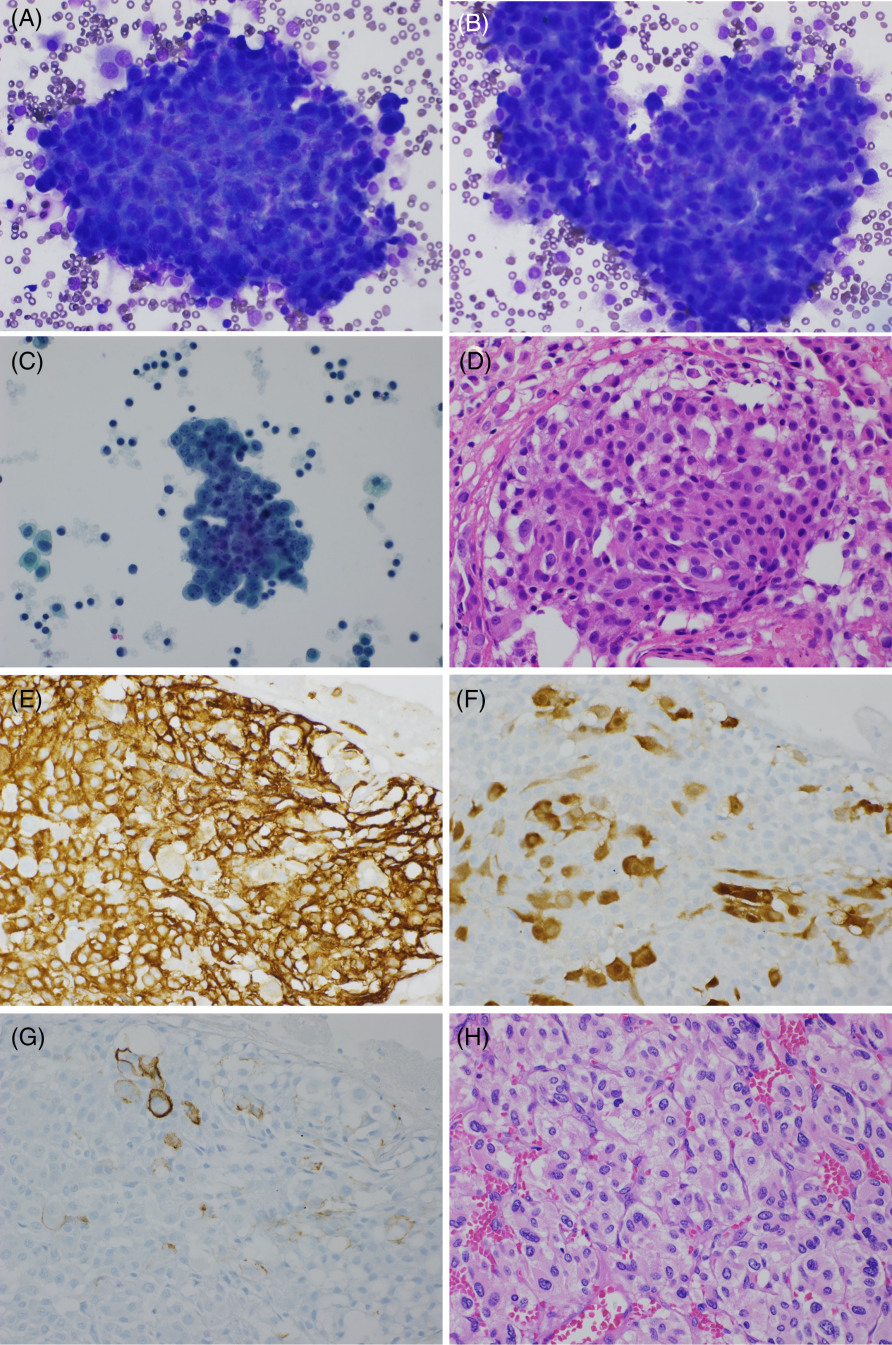
Pleural effusion with *nodular histiocytic/mesothelial hyperplasia*. (A, B) Conventional smear showing an irregular, cohesive aggregate of mononuclear cells with round and oval nuclei (DQ stain; magnification ×400); (C) Similar cellular aggregates were observed in the ThinPrep slide (Pap Stain; magnification ×400); (D) Cell block material showing a compact cellular nodule, containing a mixed population of bland round and polygonal cells, surrounded by a thin layer of fibrin (H&E stain; magnification ×400); (E) Immunohistochemical staining showing that cellular nodules are composed predominantly of CD163 positive histiocytes (Immunohistochemistry; magnification ×400); (F) Calretinin highlights the entrapped scattered mesothelial cells within these nodules (Immunohistochemistry; magnification ×400); (G) CD34 was positive on the cell membrane of a subset of scattered mesothelial cells (Immunohistochemistry; magnification ×400); (H) Histopathological examination of the resected pleural‐based mass showing a typical paraganglioma with nests of epithelioid cells separated by fibrovascular stroma (H&E stain; magnification ×400).

NHMH is a benign proliferative process that was first described in 1975 by Rosai and Dehner as a “benign reactive condition simulating a neoplastic process” in hernia sacs.^1^ Since then, NHMH has been reported in other locations where a serosal lining may be found including the lung,^2^ pleura,^3^ pericardium,^3^ pelvis,^3^ ovarian surface^3^ and urinary bladder.^4^ Among them, pulmonary sites are most frequently affected.^5^ Histologically, NHMH has been described as poorly formed, compact, nodular cellular aggregates which are primarily composed of histiocytes with entrapped scattered mesothelial cells. Naylor proposed that NHMH in serous effusions was the result of histiocytes and occasional mesothelial cells becoming enmeshed in fibrin which contacts, thereby creating compact nodules.^6^ However, there is to the best of our knowledge no recognized cytomorphologic description of NHMH. Choi et al^7^ reported a similar patient with NHMH of a pleural effusion, but without significant contributory medical history. A chest CT in their case showed a large amount of pleural fluid and multiple small nodules on the pleural surface. Their cytology sample contained vaguely nodular aggregates composed of mononuclear cells with bland morphology, entrapped mesothelial cells, and background lymphocytes. A pleural biopsy in their case report showed similar findings.

In the present case, given the CT finding of a large pleural‐based mass and cytologic appearance of NHMH in pleural fluid the differential diagnosis included malignant mesothelioma, metastatic adenocarcinoma, and less likely a soft tissue pleural lesion (e.g., solitary fibrous tumor). It is believed that NHMH results from chronic irritation of the mesothelial surface from tumor, inflammation and/or mechanical irritation. In the present case, NHMH was most likely induced by the large mediastinal paraganglioma abutting the pleural mesothelium. In accordance with a previous report of NHMH,^8^ CD34 was positive on the cell membrane of a subset of scattered mesothelial cells in our case. CD34 has been reported to participate in the cell adhesion process.^9^ Therefore, it is speculated that NHMH could be mediated by adhesion molecules and their respective ligands released after injury and cytokine stimulation,^8^ similar to the mechanism described in benign mesothelial/monocytic incidental cardiac excrescences (MICE) sometimes found during cardiac surgery.^10^

In conclusion, we report a rare case of pleural fluid NHMH in which the cytomorphology and immunoprofile are described. NHMH in body fluid cytology specimens may mimic malignant cell clusters. Awareness of this entity will help prevent diagnostic errors and unnecessary therapy.

## AUTHOR CONTRIBUTIONS

All authors were equally involved in the conceptualization, review, writing, and editing of the manuscript.

## CONFLICT OF INTEREST

The authors declare no conflict of interest.

## Data Availability

Data sharing is not applicable to this article as no new data were created or analyzed in this study.
